# High-quality reference genome sequences of two Cannaceae species provide insights into the evolution of Cannaceae

**DOI:** 10.3389/fpls.2022.955904

**Published:** 2022-07-28

**Authors:** Yuhua Fu, Sirong Jiang, Meiling Zou, Jianjia Xiao, Long Yang, Chunfang Luo, Ping Rao, Wenquan Wang, Zhengui Ou, Fanzhi Liu, Zhiqiang Xia

**Affiliations:** ^1^Guizhou Institute of Subtropical Crop, Guizhou Academy of Agricultural Sciences, Xingyi, China; ^2^Sanya Nanfan Research Institute of Hainan University, Hainan University, Sanya, China; ^3^College of Tropical Crops, Hainan University, Haikou, China

**Keywords:** Cannaceae, genome, whole-genome duplication, starch synthesis, germplasm

## Abstract

*Canna edulis* Ker-Gawl and *Canna indica* L. are species belonging to the Cannaceae family and both have a very high economic value. Here, we aimed to assemble genomes of *C. edulis* and *C. indica* at the chromosome level to generate a reference genome for the Cannaceae family. We also comparatively analyzed the genomes of *C. edulis* and *C. indica* and examined the molecular mechanisms responsible for the remarkable differences in plant characteristics in *C. edulis* varieties. Our results indicated that genome-wide duplication events had recently occurred in *C. edulis* and *C. indica*. The comparative analysis of the genomes of *C. edulis* and *C. indica* revealed that *C. edulis* exhibited a remarkable level of replication of genes in the starch and sucrose metabolic pathways, especially during sucrose hydrolysis. This finding is consistent with the fact that the starch content of the *C. edulis* tuber is higher than that of *C. indica*. Simplified genome re-sequencing revealed the population structure of 241 *C. edulis* genes, and a genome-wide association study of leaf traits revealed the location of key genes related to leaf color and morphology. These findings extend our understanding of Cannaceade at the molecular level, and provide an effective theoretical basis for further study and utilization of Cannaceae plants.

## Introduction

*Canna edulis* Ker-Gawl and *Canna indica* L. are species belonging to the Cannaceae family, with the former being a starch-rich multipurpose crop and the latter being a common herbaceous horticultural ornamental plant. Although very different regarding usage purposes, they both have a very high economic value. *Canna* is a genus belonging to the family Cannaceae. It includes approximately 51 species, of which five species, namely, *C. indica*, *C. glauca*, *C. iridiflora*, *C. warscwiczii*, and *C. flaccid*, are elementary species used to produce various hybrids that could result in the generation of an ornamental or starch-rich rhizome (Khoshoo and Mukherjee, 1970). Archaeological evidence suggests that *Canna* originated from Peru, South America (Ugent et al., 1984), and is now widely distributed in tropical and sub-tropical areas. *Canna* is a diploid (2*n* = 18) or triploid (3*n* = 27) species. Triploid *Canna* has two subtypes, namely, autotriploids and segmental allotriploids (Mukherjee and Khoshoo, 1970). Karyological analysis showed that only a single triploid type occurs in *C. discolor*, while most taxa of the genus *Canna* are diploids (Tanaka et al., 2009). *Canna edulis* is often referred to as “edible canna” (also known as “achira”) and has traditionally been used as a staple food by Andean populations for more than 4,000 years (Gade, 1966). It is commercially cultivated in Australia for starch production and in India for its edible roots (Reddy, 2015). In some tropical and subtropical regions, it is an important traditional crop that is often consumed fresh and used for starch extraction. Its fermentation could result in the production of industrial fuel ethanol (Zhu et al., 2020). A related plant of the same genus, *C. indica* L., is a model species of the family Cannaceae. It is a perennial herb that can absorb sulfur dioxide, hydrogen chloride, and carbon dioxide and beautify the environment. *Canna indica* is native to the West Indies, Malay Peninsula, and South America (Ei et al., 2015). Current studies on *C. edulis* focuses on the chemical components of *C. edulis* and the physical and chemical properties of its main component, i.e., starch (Zhang and Wang, 2013). These studies have also evaluated the utilization of various resources and the breeding of varieties. Studies on *C. indica* focus on its ability to purify heavy metals, such as copper, nickel, zinc, cadmium (Dong et al., 2019), chromium, and lead, and remove nitrogen and phosphorus from sewage water (Pinninti et al., 2021). A few studies have reported on the genomes of *C. edulis* and *C. indica*. The genetic relationship among the many cultivars of *C. edulis* is unclear. The molecular mechanism resulting in remarkable differences in plant characteristics, such as height, yield, tuber color, leaf color, vein color, and flower color in different varieties, is unclear. The mechanism by which the unique characteristics associated with the tuber height and starch production rate in *C. edulis* compared with those of *C. indica* remains unclear. Therefore, it is critical to obtain detailed genome-wide maps of *C. edulis* and *C. indica* to study their molecular breeding characteristics. The decrease in the cost of sequencing has resulted in the increased use of high-throughput sequencing for the sequencing of various plant genomes (Shangguan et al., 2013). In the present study, chromosome-scale reference genomes of *C. edulis* and *C. indica* were obtained using nanopore sequencing and Hi-C technology.

A remarkable amount of data has been obtained for *C. edulis* genomes of 241 different species found worldwide, which include 105 landraces and 136 improved cultivars. These were sequenced to further understand the genetic basis of the selection of *C. edulis*. The reproductive history of *C. edulis* was elucidated by identifying the loci and genes associated with important agronomic traits that were selected during domestication and reproduction. A genome-wide association study (GWAS) of leaf traits was performed to locate the key genes related to leaf color and morphology. Our results have provided new knowledge for the further study of molecular breeding and laid the foundation for the development of precise gene-editing techniques.

## Materials and methods

### Leaf sample collection and DNA library construction

Fresh leaves were collected from the Guizhou Subtropical Crop Institute (Guizhou, China) and frozen immediately High-quality genomic DNA was extracted from the freshly frozen leaf tissues of *C. edulis* and *C. indica* strains using a Plant Genomic DNA Kit (Tiangen), in accordance with the manufacturer’s specifications. The genomic examination and analysis of a short Illumina DNA library with an insertion length of 350 bp (137 × coverage) was performed using an Illumina HiSeq 2500 sequencer. In a Nanopore sequencing instrument, the official tool Guppy was used for base calling, and a mean_qscore_template value ≥7 was used to obtain pass reads. Subsequently, pass reads could be directly used for assembly. The *C. edulis* library had 96.5 Gb of data. Based on the statistical analysis performed following off-machine data filtering and quality control, the total volume of the off-machine data for the project after the filtering of data was 75.1 Gb, the number of reads was 3,122,701, the average read length after data aggregation was 24.0 kb, and the read length N50 was 32.5 kb; the longest read length was 290.8 kb. The total data volume obtained for *C. indica* was 94.28 Gb, the number of reads was 4,299,296, the average read length after data aggregation was 21.93 Kb, and the read length N50 was 28.84 Kb.

The assembly of three generations of reads was performed after quality control, using the Nextdeonovo software^1^ for error correction and assembly. The error correction parameters were as follows: read_cutoff = 1 k; seed_cutoff = 25 k. After error correction, the nextgraph parameters -n 73 -Q 8 -I 0.35 -S 0.24 -N 2 -r 0.62 -m 9.69 -C 1219504 -z 20 were used during assembly. Minimap2 default parameters were used to compare the three generations of data to the assembled genome. NextPolish (Jiang et al., 2020) was used to perform four continuous iterative corrections. Then, the default parameters of BWA were used to compare the second-generation data to the third-generation corrected genome data. NextPolish was used four times to make continuous iterative corrections and obtain the corrected genome sequence. Purge haplotigs (Roach et al., 2018) software was used for de-redundancy processing, for comparing the corrected genomes using the following parameters: purge_haplotigs contigcov −–15-m 72-h 190 and purge_haplotigs purge—a 98. The BioNano data solution was used as a single-enzyme cutting technology, and DLE-1 was used for digestion to obtain raw data. Certain processes were performed for quality control and filtering using the BioNano raw data, to obtain clean data for subsequent analysis. BUSCO (Waterhouse et al., 2017) was used for the prediction of the genome sequence; a single copy of the homologous gene in the plant library (embryophyta_odb10) was used to predict the gene status of the existing sequence in the genome. Hi-C libraries were built and sequenced on the Illumina NovaSeq platform, and a chromosome framework was created. Hi-C Libraries were analyzed *via* the Juicer Pipeline (Durand et al., 2016) and visualized using Juicebox.^2^ In summary, the genome exhibited nine pseudochromosomal molecules and 239 non-localized structures. Genome assembly data have been deposited in the BIGD database under the project ID number PRJCA007548.

### Genomic annotation

Repeat sequence prediction was based on sequence self-alignment. The software RepeatModeler (Flynn et al., 2020; version 1.0.4, https://github.com/rmhubley/RepeatModeler) was initially used to establish the *de novo* repeat sequence library, and then the Repeatmasker (version 4.0.5, http://www.repeatmasker.org/) software was used for performing predictions. Gene structure prediction was based on a combination of evidence-based prediction and *ab initio* prediction (*de novo*). The Maker software and the subsequently generated evidence files were used to perform the evidence-based assembly. The evidence files contained (ncbishangx) RNA-seq data, obtained through Hisat2^3^ alignment, Trinitty (Grabherr et al., 2011; https://github.com/trinityrnaseq/trinityrnaseq/wiki) assembly, and Cufflinks (Trapnell et al., 2012; http://cole-trapnell-lab.github.io/cufflinks/), along with de-redundant transcripts and protein sequences of related species. In addition, Augustus (Mario et al., 2006; http://bioinf.uni-greifswald.de/augustus/) was used for *de novo* annotation. Then, the results of Maker and *de novo* analyses were integrated based on the principle that the obtained result is better than the predicted result. Then, the results of the integration process were filtered to exclude proteins in which repetitive sequences composed more than 50% of the protein or proteins with lengths less than 50 aa. The gene set was further screened based on gene expression and near-source species support to obtain the final gene set. Direct homology was identified based on sequence similarity. The source gene, through a protein with a known function, helps to infer the function of the new gene. The eggNOG-mapper (Cantalapiedra et al., 2021) and BLAST software were used to annotate genes in the gene ontology (GO), Kyoto Encyclopedia of Genes and Genomes (KEGG), clusters of orthologous genes (COG), non-redundant (NR) database, Pfam, and other databases.

### Gene family classification and comparison

Protein and nucleotide sequences from *C. edulis*, *C. indica*, and other sequenced plant genomes (*Musa acuminata* and *Ensete* Bruce ex Horan), monocotyledonous plants (*Elaeis guineensis* Jacq, *Phoenix dactylifera* L, *Zea mays* L, *Setaria italica*, and *Ananas comosus* L. Merr), and dicotyledonous plants (*Cannabis sativa*, *V. vinifera*, *Carica papaya*, *Arabidopsis thaliana*, and *Amborella trichopoda*) were used to construct gene families using OrthoFinder.^4^ We performed KEGG and GO annotation of genes using KOBAS software. Single copies of genes from *C. edulis*, *C. indica*, and four other angiosperms (*A. trichopoda*, *A. thaliana*, *M. acuminata*, and *V. vinifera*) were used to identify single-copy genes using OrthoVenn2.

The rRNAs were predicted using RNAmmer (Lagesen et al., 2007; version 1.2), while tRNAs were predicted using tRNAscan-SE (Lowe and Eddy, 1997; version 1.23). Other ncRNA sequences were identified using the Perl program Rfam_scan.pl. (version 1.0.4) *via* inner calling using Infernal (Nawrocki and Eddy, 2013; version 1.1.1). The iTAK (Zheng et al., 2016) software was used to detect known TFs in the *C. edulis* and *C. indica* genomes and other plants.

CAFE (version 3.0; Tijl et al., 2006) was used to determine the expansion or contraction of gene families. Gene families of at least five species were selected for further analysis. Variabilities in gene families were assessed by examining random birth and death patterns in each row of the phylogenetic tree. The probability of each gene family migrating from parent to child during phylogeny was calculated using a probabilistic graphical model by analyzing the expansion and contraction of the family across all nodes and types.

### Genome duplication analysis

Whole-genome Duplication Integrated (WGDI; Sun et al., 2021) analysis was used to detect collinear regions using the all-to-all BLASTP results (at least 10 pairs of homologous genes in a block, less than five genes in the null assay) for *C. edulis*/*C. edulis*, *C. indica*/*C. indica*, *C. edulis*/*C. indica*, banana/*C. indica*, banana/*C. edulis*, grape/*C. indica*, and grape/*C. edulis*.

The *K*s values of homologous gene pairs were calculated with WGDI (version 4.8) using the YN00 NG model. To identify duplicated genes in *C. edulis* and *C. indica*, we used the MCScan (Wang et al., 2012) software module Duplicate_gene_classifier, to classify duplicated/WGD fragments, and tandem, proximal, or dispersed duplications. Other genes were defined as singletons. Then, WGD genes/genes with repeated segments were divided into two sub-categories. Gene pairs with a *K*s value of 0.3–0.4 on the same line were defined as modern reserved WGD genes, and the others were identified to be old reserved WGD genes.

### Population genetic analysis

Qualified next-generation sequencing reads from 241 *C. edulis* germplasms were mapped to the *C. edulis* genome using the BWA software (version 0.7.17; Li, 2013). The original genome re-sequencing data were stored in the BIGD database.

Each genome was aligned by labeling duplicate reads with the Picard tool,^5^ and indel reads were realigned using the Genome Analysis Toolkit (GATK) using RealignerTargetCreator and IndelRealigner. Variant calling of each genome was performed using the GATK HaplotypeCaller to generate variant call format (VCF) files. The original VCF file was deposited in the BIGD database under project ID number PRJCA007550. All the VCFs for the 241 genomes were merged into a single VCF file using the GATK Genotype GVCF function (version 4.1.8.1). The VCFtools (Danecek et al., 2011) software was used to filter out SNPs. SNP annotation was conducted based on the *C. edulis* genome. The snpEff (Cingolani et al., 2012) software was used to classify the positions of SNPs into spacers, untranslated regions (including 3′ and 5′ untranslated regions), intron regions, and codon regions.

### Population structure and genetic diversity analysis

The genetic distance matrix of samples was first calculated using PHYLIP.^6^ The family tree file was built using the Neighbor software. Phylogenetic tree diagrams were drawn using iTOL. The principal components of the *C. edulis* population were analyzed using the GCTA software (Yang et al., 2011), in which SNPs were identified as input data. Then, the R software was used to calculate each principal component vector and draw a PCA scatter plot. Additionally, the ADMIXTURE (Alexander et al., 2009) software was used to determine the number of subgroups in the population accurately. PLINK (Purcell et al., 2007) software could process VCF files and convert files in the VCF format into files in various formats. The range of *k* values for subgroups was fixed at 1–9. Based on the CV error value obtained, the appropriate *K* value for the number of subgroups was determined. The genetic composition factor Q of each substance in each subpopulation was used to construct a matrix of population genetic structure. In the whole group and each subgroup, 39,980 SNP markers were compared in pairs using the *R*^2^ value for the entire genome.

### Linkage disequilibrium analysis

In the total population and each subpopulation (inferred using ADMIXTURE), we used the PopLDdecay (Zhang et al., 2019) software and high-quality SNPs after the filtering process, calculated the *R*^2^ value, and assessed the linkage disequilibrium (LD) association between each pair of polymorphic loci in the genome. The LD distances were sorted from small to large, and the mean value of *R*^2^ was calculated within the segment to plot a smooth curve scatterplot. When the smooth curve decays to a certain size, the corresponding physical distance is equal to the distance of LD decay.

### Association analysis

In this study, 39,990 high-quality SNP and indel data were used as the input to perform a GWAS analysis to assess the leaf shape and color in this population. A compressed MLM of TASSEL 5.0 (Bradbury et al., 2007) software was used during correlation analysis.

The significance limit of the tolerance level was set at 0.05. The physical location and value of the SNPs in the *C. edulis* genome were entered, and the qqman R software package was used to draw a QQ and Manhattan map. Regions significantly associated with the recombination results for the *C. edulis* reference genome were manually examined using SAMtools.

## Results

### Genome assembly and annotation

**Figure 1:**
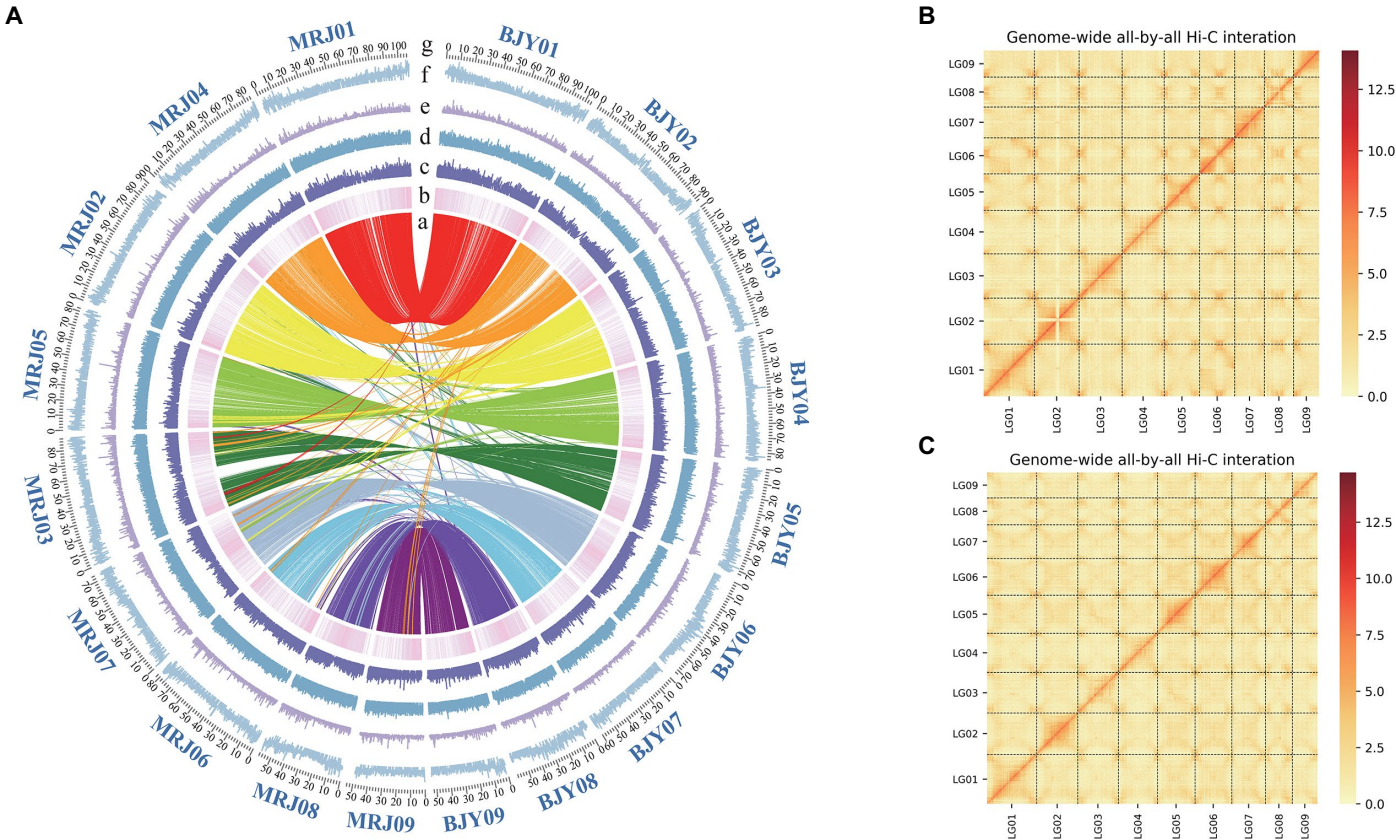
**(A)** Genomic feature display. **(a)** The collinearity between the *Canna edulis* and *Canna indica* genome **(b)** Gene density **(c)** RNA-seq expression **(d–f)** Different repeat densities DNA, LINE, and LTR. **(B)** Chromosome interaction map for the *C. edulis* genome. **(C)** Chromosome interaction map for the *C. indica* genome. **(g)** The number and length (Mb) of pseudochromosomes are indicated outside of the ring.

The genome sizes of *C. edulis* and *C. indica* were estimated to be 844.9 and 836.88 Mb, respectively, using k-mer analysis (Supplementary Table 1). Using the Nanopore Technology system (Supplementary Table 2), a total of 31.23 million nanopore-cleaned reads (~75.1 GB of data, ~93x coverage) and 4.3 million nanopore-cleaned reads (~94.28 GB data, ~100x coverage) were obtained. The genomes were assembled using NextDenovo. The final assemblies were 823.4 Mb in size (contig N50 length, 17.3 Mb; longest contig, 52.8 Mb) for *C. edulis* and 834.9 Mb (contig N50 length, 2.1 Mb; longest contig, 31.9 Mb) in size for *C. indica* (Table 1). The contigs and scaffolds of *C. edulis* and *C. indica* were further scaffolded into nine chromosomes using Hi-C technology, and the sizes of the anchored genomes were 691.7 Mb (84.69%) and 725.7 Mb (87.15%), respectively (Figure 1; Table 1). The assembled genomes were more than 96.87 and 96.73% complete (Supplementary Table 3), as shown *via* Benchmarking Universal Single-Copy Orthologues (BUSCO) analysis. The LTR Assembly Index (LAI) was simultaneously used to evaluate the genome assembly continuity. The LAI values of *C. edulis* and *C. indica* were 10.2 and 12.46, respectively. The corresponding second-generation genomic data were compared with the genome data *via* Burrows-Wheeler Aligner (BWA). The rates of similarity between the genome data and second-generation data were 99.79 and 99.78%, respectively. Therefore, the level of data integrity after the assembly of the two genomes was high.

**Table 1:** Statistics for *Canna edulis* and *Canna indica* genome assembly.

Assembly and annotation feature	*Canna edulis*	*Canna indica*
Assembly size(bp)	823,408,298	834,891,454
Contig N50 size(bp)	17,301,877	2,100,650
Number of contigs	398	699
LAI complete percentage in assembly	10.2	12.46
BUSCO complete percentage in assembly (%)	96.87	96.73
Predicted protein-coding genes	30,578	32,957

The prediction of protein-coding genes was performed on the basis of a combinatorial approach, homology, and transcripts. A total of 30,578 *C. edulis* and 32,957 *C. indica* protein-coding genes were identified. The average gene lengths, exon numbers, and coding sequence lengths were 3572.73 bp, 4.6, and 227.618 bp, respectively, for *C. edulis*, and 2,759 bp, 4.3, and 224.927 bp, respectively, for *C. indica*. These values are similar to those for other *Musaceae* crops, such as the banana plant. A total of 240 and 209 miRNAs, 1,818 and 2,028 tRNAs, 815 and 742 snRNAs, and 12,486 and 4,803 rRNAs were found in the *C. edulis* and *C. indica* genomes, respectively. More than 62.62 and 63.60% of the *C. edulis* and *C. indica* genomes, respectively, were found to be composed of repetitive elements that were similar to those of maize (~64%), and the number of repetitive elements was higher than that in banana (~55.75%). The proportions of different types of repeats were remarkably similar in the two genomes. Among these, long terminal repeats (22.80 and 24.15% for *C. edulis* and *C. indica*, respectively) represented the largest proportion of repeats, followed by DNA transposons, (9.63 and 10.59% for *C. edulis* and *C. indica*, respectively), and long interspersed nuclear elements (3.46 and 3.26% for *C. edulis* and *C. indica*, respectively; Supplementary Table 4) represented the smallest proportion of repeats. This finding is similar to the findings observed for the bamboo and banana plants. Additionally, 1,859 and 1,918 transcription factors were identified in the *C. edulis* and *C. indica* genomes, respectively, using the iTAK software (Supplementary Table 5).

### Construction of the gene family

**Figure 2:**
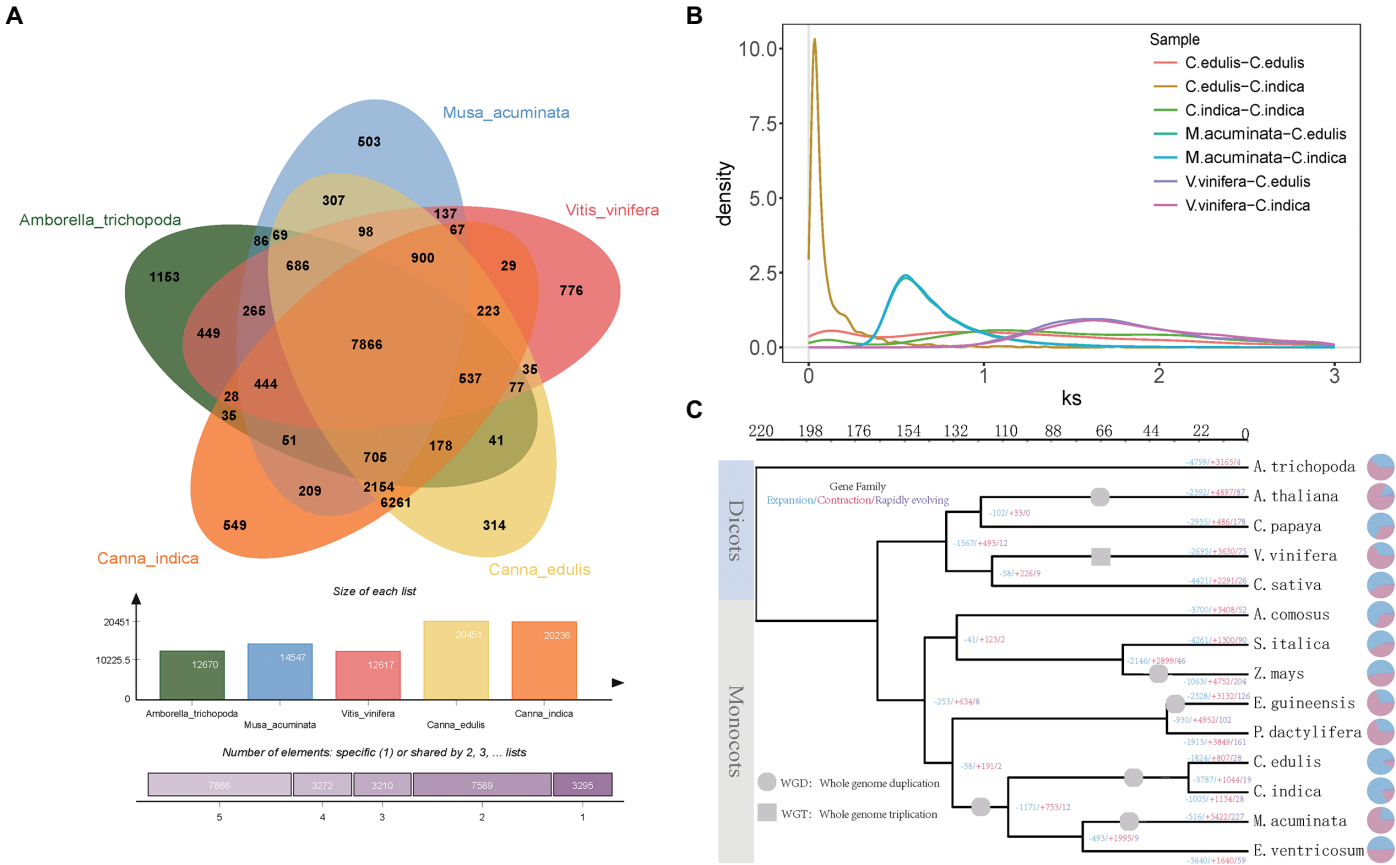
**(A)** Analysis of gene families of five species (*Canna edulis*, *Canna indica*, *Amborella trichopoda*, *Musa acuminata*, and *V. vinifera*). **(B)** Frequency distribution of synonymous substitution rates (Ks) between homologous gene pairs in syntenic blocks of *C. edulis*-*C. edulis*, *C. indica*-*C. indica*, *C. edulis*-*C. indica*, *M. acuminata*-*C. edulis*, *M. acuminata-C. indica*, *V. vinifera*-*C. edulis*, and *V. vinifera*-*C. indica*. **(C)** Phylogenetic tree of 14 species constructed using single-copy orthologous genes.

It was found that a total of 241 gene families and 287 gene families were specific for *C. edulis* and *C. indica*, respectively (Figure 2A). The Venn diagram displayed in Figure 2A shows that *C. edulis*, *C. indica*, *Amborella trichopoda*, *M. acuminata*, and *V. vinifera* have a total of 7,866 gene families. Genes encoding five genomic histone proteins (Supplementary Table 6) were grouped into 47,186 gene families (two or more), of which 7,866 gene families were common to all families and 67 gene families were restricted to legumes (Cannabidiaceae). Among the Cannaceae-specific gene families, only 314 and 549 were unique to *C. edulis* and *C. indica*, respectively (Figure 2A). In *C. indica*-specific gene families, genes related to oxidative phosphorylation; metabolic pathways; biosynthesis of secondary metabolites; biosynthesis of unsaturated fatty acids; amino sugar and nucleotide sugar metabolism; ascorbate and aldarate metabolism; and photosynthesis and fatty acid metabolism were remarkably enriched. In *C. edulis*-specific gene families, genes related to cyanoamino acid metabolism; metabolic pathways; starch and sucrose metabolism; isoquinoline alkaloid biosynthesis and phenylpropanoid biosynthesis were remarkably enriched (Supplementary Table 7).

### Genome evolution

The evolutionary relationships of *C. edulis* and *C. indica* with other sequenced plant genomes (*M. acuminata* and *Ensete* Bruce ex Horan), monocotyledonous plants (*Elaeis guineensis* Jacq*, Phoenix dactylifera* L, *Zea mays* L, *Setaria italica*, *Ananas comosus* L. Merr), and dicotyledonous plants (*Cannabis sativa*, *V. vinifera*, *Carica papaya*, *Arabidopsis thaliana*, and *Amborella trichopoda*) were examined. Phylogenetic analysis was performed (Supplementary Table 8). The results obtained for the link-based targeting of 37 single-copy gene families from 14 sequenced plant genomes support this system of *C. edulis* and *C. indica*, along with that of *M. acuminata* and *Ensete* Bruce ex Horan in the Cannaceae family (Figure 2C). The results showed that the differentiation of *C. edulis* and *C. indica* occurred at a later period, at approximately 30–44 MYA, and their common ancestor was 140 MYA away from grass species. The improved informative trait sample should allow for a more accurate assessment of the differentiation period during genome-wide phylogenetic analysis.

Pedigree analysis shows that Cannaceae and Musaceae are closely related to each other, and the estimated divergence between Cannaceae and Musaceae occurred at about 120 MYA (110–132 MYA) in the Scitamineae branch.

In this study, whole-genome duplications (WGDs) in *C. edulis* and *C. indica* were analyzed for homology, *via* a self-comparison of the encoding genes in *C. edulis* and *C. indica*. The results strongly support the recent occurrence of separate WGD events in the *C. edulis* and *C. indica* genomes. The Ks values for *C. edulis* versus *C. edulis*, *C. indica* versus *C. indica*, *C. edulis* versus *C. indica*, *M. acuminata* versus *C. edulis*, *M. acuminata* versus *C. indica*, *V. vinifera* versus *C. edulis*, and *V. vinifera* versus *C. indica* collinear orthologs indicated that the WGD event occurred before the split of Cannaceae and Musaceae and after the split of Cannaceae and *V. vinifera*. The splitting of *C. edulis* and *C. indica* occurred after the occurrence of a specific WGD event (Figure 2B).

**Figure 3:**
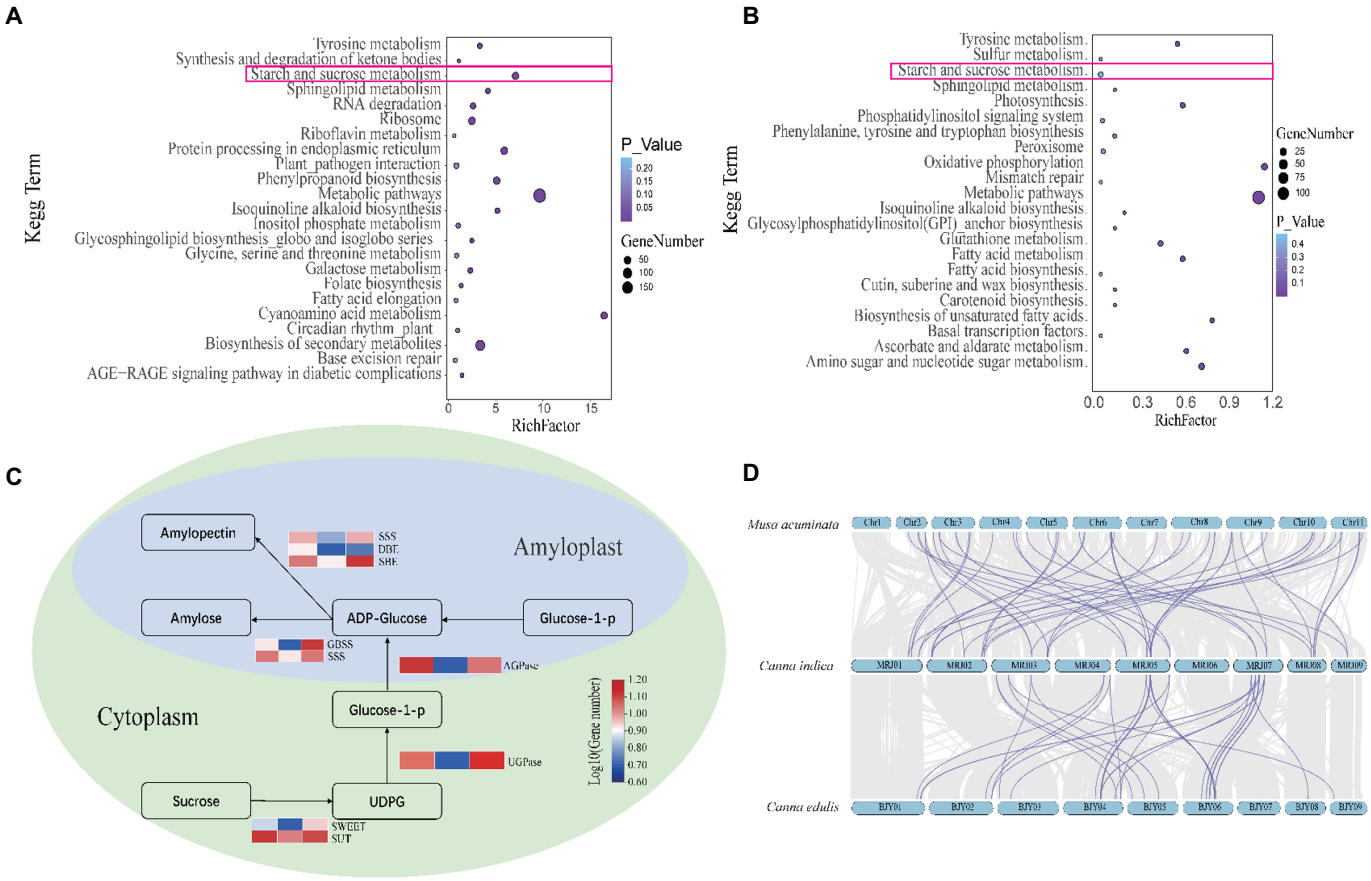
**(A)** Kyoto Encyclopedia of Genes and Genomes (KEGG) analysis of unique genes in *Canna edulis*. **(B)** KEGG analysis of unique genes in *C. indica*. **(C)** Comparison of gene copy numbers of *C. edulis*, *C. indica*, and *M. acuminata* in the pathways for starch and sucrose metabolism. **(D)** The gray area represents the collinearity block of *C. edulis*-*C. indica* and *M. acuminata*-*C. indica*. The purple line indicates that *C. edulis* and *M.acuminata* exhibited significantly expanded gene pairs, as compared to *C. indica*.

Amongst the 2,659 gene families identified in the genome of *C. edulis*, 807 were significantly expanded (*p* < 0.05), and 1,824 were contracted (*p* < 0.05). Amongst the 2,167 gene families detected in the *C. indica* genome, 1,134 were expanded, and 1,005 were contracted. The KEGG pathway analysis of the expanded gene families in *C. edulis* revealed marked enrichment of genes involved in cyanoamino acid metabolism. It also showed that genes related to cyanoamino acid metabolism; phenylpropanoid biosynthesis; starch and sucrose metabolism; biosynthesis of secondary metabolites and sphingolipid metabolism; and metabolic pathways were significantly enriched. The KEGG pathway analysis of the expanded gene families in *C. indica* revealed a marked enrichment of genes involved in metabolic pathways; biosynthesis of secondary metabolites; alanine, aspartate, and glutamate metabolism; glycine, serine, and threonine metabolism; fatty acid degradation and glycolysis/gluconeogenesis; and tyrosine metabolism (Supplementary Table 9). A comparison of the KEGG enrichment results of *C. edulis* and *C. indica* showed that the starch and sucrose metabolic pathways occurred more prominently in *C. edulis*. Therefore, the genes number in this pathway was compared. The results showed that the number of genes related to starch synthesis was significantly higher in *C. edulis* than in *C. indica*, which was consistent with the higher abundance of starch content in the root tuber of *C. edulis* than in the root tuber of *C. indica*. This study focused on the starch and sucrose metabolism pathway. During transient starch synthesis, the Calvin cycle enables the fixation of CO_2_ and formation of 3-phosphoglyceric acid, which is converted into triose phosphate (TP) and transported *via* TP translocation. In the cytosol or the chloroplast, TP is converted into fructose 6-phosphate, and then into glucose 6-phosphate and glucose 1-phosphate (G1P). G1P facilitates the formation of adenosine diphosphate glucose (ADPG) under the influence of ADPG pyrophosphorylase (AGPase). The branching enzyme (BE) and debranching enzyme (DBE) are used to synthesize amylose and amylopectin. The synthesis of sucrose synthase (SuSy) enables organic matter that has been fixed *via* the photosynthesis of leaves to be transported to the organs involved in starch synthesis in the form of sucrose, and enables the conversion of the organic matter into G1P, which successively forms amylose and amylopectin in the presence of AGPase, SuSy, BE, and DBE. The present study found that genes related to starch synthesis were remarkably expanded in *C. edulis*. A comparison of the *C. indica* genome with the published genome of *M. acuminata* showed that *C. indica* also exhibited a trend of remarkable expansion of the starch and sucrose metabolism pathways. A total of 174 genes involved in starch synthesis were identified in *C. edulis*, and only 72 were identified in *C. indica*. Among these genes, *SWEET* and *SUT* occurred in the largest proportion (Supplementary Table 10; Supplementary Figure 1), which indicates that the process of sucrose transport plays an extremely important role in the accumulation of starch in the roots of *C. edulis*. This finding provides a theoretical basis for the genetic improvement of *C. edulis*. Notably, the expansion of these genes, especially those associated with starch and sucrose metabolism, and the biosynthesis of secondary metabolites and metabolic pathways could contribute to starch accumulation, plant growth, and the biological adaptability of the species (Figure 3).

### Genotype analysis of the *Canna edulis* population

A total of 155 G of data were obtained through Hyper-seq (Zhiqiang et al., 2021) database construction and the sequencing of 241 *C. edulis* genomic DNA sequences, and 134 G of data and 893,020,018 reads were obtained after the filtering of data. The assembled plantain genome was reused as the reference genome. A total of 15,659,890 SNPs and indels were obtained. We used vcftools to filter all single nucleotide polymorphisms (SNPs), insertions, and deletions (indels), including MAF (Minor Allele frequencies) and HWE (Hardy–Weinberg equilibrium), and retain 39,980 high-quality SNPs and 2,107 indels from the original file. High-quality SNP annotation results showed that 36,790 SNPs (69.14%) occurred in the intergenic regions and 13,166 SNPs (24.74%) were found in genomic regions. In the coding region, three SNPs produced silent mutations, and 1,679 SNPs produced missense mutations (Supplementary Table 11).

### Analysis of the population structure of *Canna edulis*

**Figure 4:**
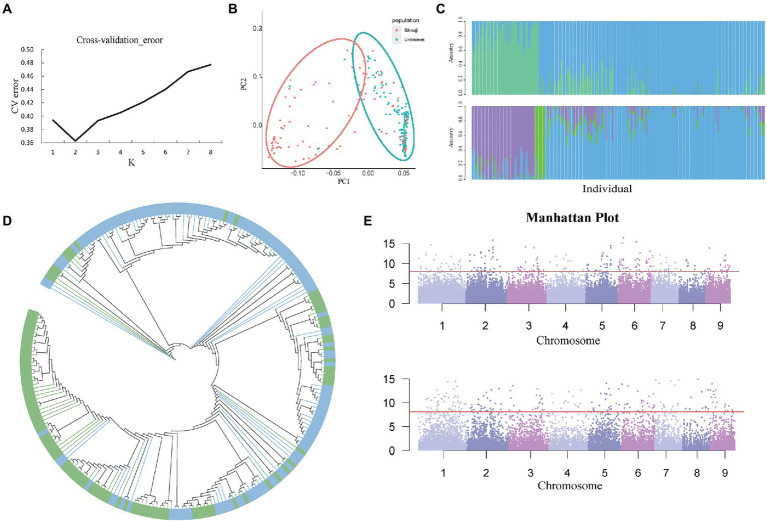
**(A)** Cv-error value under different *K* values. **(B)** PCA analysis based on 241 *C. edulis* samples. **(C)** The group structure of *C. edulis* when *K* = 2 or 3. Different colors represent different subgroups. **(D)** The neighbor-joining tree of the *C. edulis* population was constructed based on genetic distances; the blue color represents subgroup1, and the green color represents subgroup2. **(E)** Genome-wide association study (GWAS) analysis of two traits of leaf shape and color of *C. edulis*.

Population structure analysis was performed for all 39,980 high-quality SNPs using the admixture software. The maximum number of clustered subgroups (*K*) was inferred to range from 1 to 8, and the cross-validation error rate was calculated at each value of *K* (CV error); when the *K* value increased from 1 to 2, the CV error decreased rapidly, and if *K* is greater than 2, the CV error gradually increased and becomes constant (Figure 4A). Therefore, when *K* is equal to 2, the CV error is the smallest, indicating that a value of *K* = 2 is the most suitable, i.e., the entire rice population is divided into two subgroups, namely subgroup 1 and subgroup 2. In R software, we set a value of *K* = 2, which resulted in the classification of the population into two subgroups. Then, we performed a *K*-means cluster analysis of the filtered original SNP database and obtained the *K* value matrix. Calculate the genetic distance of the population, and the genetic distance was imported into the Neighbor software to obtain a tree file. Then, the two subgroups were classified and shown in two different colors, and the phylogenetic tree was visualized in iTOL, and the results of clustering analysis were obtained (Figure 4B). After the analysis was completed, an image of the population structure was generated by R software using the two subgroups inferred by ADMIXTURE from the group. We found that PCA could enable the two subgroups to be classified into two groups as they could be distinguished on the PC1 axis.

The *Q* values for each of the materials in the two subgroups were calculated using ADMIXTURE software (Figure 4C). Subgroups 1 and 2 had 104 and 137 germplasm resources, respectively. Although the two subpopulations were distributed differently on the PC1 axis, the results of the summation process are consistent with the distribution of the population structure. However, the two subgroups of *C. edulis* are not clustered together effectively on the phylogenetic tree. Some crossover was observed between samples. The results of the study showed that there was no significant correlation between the genetic relationships in the *C. edulis* population and the geographical origin (Figure 4D).

### GWAS of *Canna edulis* germplasms

*Canna edulis* has a diverse variety of leaves. In terms of morphology, curly and flat leaves can be observed. Leaves are classified as fully green leaves, fully purple leaves, and green leaves with purple fringes, based on their color. Data on these traits were collected for GWAS analysis with genome-wide markers for SNP diversity. Simple templates and a compressed mixed linear model (MLM) were used to identify relevant signals. As shown in the quantile-quantile (QQ) plot, the condensed MLM method significantly reduces the extent of false positives. This method takes the genetic relationship pattern of the entire genome into consideration. A total of 550 loci related to leaf shape traits and 240 loci related to leaf color traits were identified with *p* < 3.85 × 10^−5^ using the compressed MLM method (Supplementary Table 12; Figure 4E). The results obtained using the QQ Atlas show that the SNP sites associated with significantly correlated parameters were reliable (Supplementary Figure 3).

### *Canna edulis* genome-wide linkage disequilibrium

The linkage disequilibrium (LD) decay curves of 241 *C. edulis* germplasm resources were analyzed using 39,980 SNP markers that were recovered from the whole genome (Supplementary Figure 2). The results showed that the extent of LD decreased gradually with an increase in the physical distance between SNPs. When the attenuation threshold was set at *R*^2^ = 0.3, the attenuation distance of the entire population was about 400 kb, which is much higher than that for cultivated rice (123 kb) and cultivated corn (30 kb; Traut, 1994).

### Candidate genes

Several genes were located. Twenty-five candidate genes were identified using LD. Most candidate genes were located within mutation sites and could be up to 20 kb long (Supplementary Table 13). The genes associated with leaf shape mostly belonged to the *TCP* family of genes, which encode a subunit of chloroplast chaperonins involved in mediating the folding of newly synthesized, translocated, or stress-denatured proteins. Some *ARF* gene families, which encode one of the chloroplast/plastid-localized GAPDH isoforms (GAPCp1/At1g79530 and GAPCp2/At1g16300), were also mapped. The double GAPCp mutant shows remarkable phenotypes, such as those associated with impaired root development, dwarfism, and infertility. GAPCps are important for the synthesis of Wurzelserin. The genes associated with color development mostly belong to the *MYB* family of genes, which encode a leucine-rich repeat receptor kinase located in the cell membrane and are involved in brassinosteroid signaling. The BRI1 ligand is a brassinolide that binds to the extracellular domain. This could lead to the phosphorylation of the kinase domain, which activates the BRI1 protein in response to brinzolamide. Some genes belonging to the *TPS* gene family were identified. These genes encode a leucine-rich repeat receptor kinase involved in the occurrence of phytosulfokine, which is a five aa tyrosine-sulfated peptide that promotes cellular proliferation and encodes a protein that interacts with phytosulfokine, a five amino acid-long sulfated peptide that exhibits the catalytic activities of both diguanylate cyclase and kinase.

## Discussion

The primary goal of the genome sequencing project was to obtain a high-quality reference genome and identify a set of high-confidence SNPs from well-aligned re-sequencing data. The Cannaceae genome is relatively complex, with a high level of heterozygosity and numerous repetitive sequences. This makes it challenging to assemble the Cannaceae genome. *Canna edulis* and *C. indica* belong to the Cannaceae family but are cultivated for remarkably different purposes. Genomic studies on these two species could provide a reference for the changes in the size of the Cannaceae genome. At present, there is a lack of relevant research on the Cannaceae genomes prevalent in China. Previous studies on the genomes of the *C. edulis* and *C. indica* species were focused only on one chloroplast genome (Zhu et al., 2020). Related species with a size of 492 Mb, of which 430 Mb was mounted on 11 chromosomes, have been reported in the banana genome of the same order as the genus *Musa* (Wang et al., 2019). Third-generation nanopore sequencing techniques and NextDenovo assembly were used along with Hi-C-assisted assembly technology to obtain the chromosome-level genomes of *C. edulis* and *C. indica*. Using BUSCO, the scaffold N50 lengths of *C. edulis* and *C. indica* were determined to be 73.4 and 84.7 Mb, respectively, while the annotated gene integrity for *C. edulis* was 96.87%, and that for *C. indica* was 96.73%. The genomes of *C. edulis* and *C. indica* are highly heterozygous and have many repetitive sequences that pose a challenge to genome assembly. Therefore, we aimed to achieve more accuracy and completeness of genome assembly with the use of the Hi-C auxiliary system. The chromosome-scale reference genomes of *C. edulis* and *C. indica* provided information on the gene distribution and characteristics, repetitive elements, and genomic structure of DNA occurring on the nine chromosomes. The genomic data obtained in this study represent a valuable genetic resource for molecular and applied research for *C. edulis* and *C. indica* development. This data could also provide ideas for further research on genomic evolution and comparative genomic studies of Cannaceae and related species.

A single-copy phylogenetic analysis of 39 homologous sequences in 13 typical seed plant genomes showed that *M. acuminata* and *Ensete ventricosum* were related to each other. Together, they formed a sister group consisting of *E. guineensis* and *P. dactylifera*. Phylogenomic studies are based on the analysis of orthologous genes. In recent years, the emergence of a new generation of DNA sequencing technologies has significantly improved the range and efficiency of the sequencing process. The output resulting from the use of these techniques has become an important data source for conducting phylogenetic studies using large-scale genetic or genomic data. While analyzing the process of biological evolution, it is crucial to know the rate of occurrence of synonymous and non-synonymous mutations in a species. The current process of genome assembly improved our understanding of the timing of occurrence of WGD events in Cannaceae. The collinear analysis and Ks distribution analysis of the genome confirmed the occurrence of WGD events in Cannaceae. According to the TimeTree website,^7^ a WGD event would occur at around 40 MYA. A recent study of the evolution of the banana genome suggests that this WGD event might have occurred before the splitting of the banana and *C. edulis* genomes.

Although *C. edulis* and *C. indica* belong to the Cannaceae family and have extremely similar shapes, the *C. edulis* root tuber is rich in starch. Therefore, people attach more importance to the development and production of the *C. edulis* root tuber. A method for producing bioethanol was previously studied using starch and non-starch polysaccharides occurring in the *C. edulis* root tuber (Huang et al., 2013). The starch content in the *C. indica* root tuber is low; thus, people have not conducted much research on the development of the *C. indica* root tuber. Many studies have been conducted on the development of starch-rich crops, such as potatoes, corn, and cassava. However, *C. edulis* starch has unique characteristics. For example, it has very large rectangular particles (about 45–52 μm long on the long axis and 33–34 μm long in the short axis). The amylose content is high (approx. 33–39%). In comparison to other starch gels, the San Gaban Achira ecotype-formed gel is cooled during RVA (Rapid Visco Analyser) testing (5% strength) and consistency analysis (8% strength), to produce a gel with high levels of consistency and heat resistance (Cisneros et al., 2009). Previous studies have examined the physicochemical and structural properties of the natural starch obtained from *C. edulis* and confirmed that this starch could be used as a substitute for traditional corn and potato starch. In addition, their amylose content makes them a potential source of resistant starch and dietary fiber (Fonseca-Santanilla and Betancourt-López, 2021). Previous studies have proved that the number of copies of starch synthesis-related genes in starch-rich plants has increased significantly (Sahu et al., 2020). We arrived at the same conclusion in this study after performing gene enrichment analysis for unique genes in *C. edulis* and *C. indica*. The results showed that genes in *C. edulis* are remarkably enriched in the starch and sucrose metabolism pathways. In addition, even the KEGG analysis of the genes that were amplified significantly in the two genomes showed that in *C. edulis*, genes were remarkably enriched in the starch and sucrose metabolism pathways. This result was consistent with the difference in root starch content between *C. edulis* and *C. indica*. This difference is attributable to the difference in the number of genes in the starch and sucrose metabolism pathways. Biosynthesis can be studied most effectively in non-photosynthetic organs that store starch, such as seeds and tubers. Baroja-Fernández et al. revealed that SuSy (Sucrose Synthase) is a potent determinant of glucose, ADPG, starch, and total yield in potato tubers. The enhancement of SuSy activity is an effective strategy for increasing starch accumulation and potato tuber yield (Baroja-Fernández et al., 2009).

Zhang et al. verified the expression of 21 hypothetical unigenes encoding the major enzymes involved in starch and sucrose metabolism *via* qPCR, using RNA from chestnut seeds at five stages of growth (Zhang et al., 2015). No relevant research has been conducted on the pathways for the metabolism of starch and sucrose in *C. edulis*. The process of starch accumulation in *C. edulis* has still not been understood thoroughly. It is important to use genomic comparative analysis to analyze the pathway for starch production in *C. edulis*, to achieve the breeding and quality improvement of *C. edulis*.

The Hyper-seq re-sequencing of traditional *C. edulis* varieties showed numerous continuous polymorphisms and high-resolution correlations. However, the rate of degradation of *C. edulis* LD was very slow. This method has been successfully applied to other crops, such as *M. esculenta* Crantz (Zhang et al., 2018) and *Solanum tuberosum* L. (Wang et al., 2021). A detailed understanding of the demographic structure of a group of members is essential to exclude unnecessary members from the group. In our study, the CV error was used to determine a *K*-value of 2. The highest probability value of the membership threshold was used to classify the panel into 1–2 subgroups. Nevertheless, PCA was unable to identify significant differences on the PC1 axis, suggesting that the *C. edulis* population was composed of numerous mixed lineages and a low-level population structure.

The leaves of *C. edulis* are of diverse varieties. A GWAS analysis was conducted to locate the genes related to leaf development. This provided a theoretical basis and data resource for the future selection of *C. edulis* varieties. GWAS could identify many candidate genes and accurately identify specific tissue expression profiles in different ethnic groups. For example, Liu et al. (2020) used 584 rice germplasm materials, genotyped the materials using 700,000 SNP markers, and identified 27 loci related to rice blast resistance using GWAS. These traits could also help us to identify the locus of a high-resolution quantitative trait and understand the gene regulatory network of the trait more effectively.

In this study, the potential for GWAS was limited because of the low genetic diversity and small population sizes of *C. edulis* samples. The *C. edulis* varieties used in the new Hyper-seq sequence must be harvested globally. An extensive phenotypic analysis is currently underway, and the relationship between large samples might be investigated in the future. Genome sequencing allows the identification of new SNPs and results in better reduction efficiency under low sequence coverage; thus, GWAS could be used to effectively analyze the resolution of the map and identify new alleles *via* continuous population expansion. These studies have laid a foundation for long-term cooperation that would enable us to identify valuable genes and alleles from global germplasm resources and facilitate the improvement of plant varieties.

## Data availability statement

The datasets presented in this study can be found in online repositories. The names of the repository/repositories and accession number(s) can be found in the article/Supplementary material.

## Author contributions

ZX, ZO, and FL designed and supervised the project. LY, CL, and PR prepared the samples. SJ and JX analyzed the data. SJ wrote the manuscript. MZ and YF revised the manuscript. All authors contributed to the article and approved the submitted version.

## Funding

This work was supported by projects for the transformation of Guizhou Science and technology achievements [(2021) general 009], integrated demonstration of key techniques for the industrial development of featured crops in rocky desertification areas in the Yunnan-Guangxi-Guizhou provinces (SMH2019-2021), the “liangjiangyihe” tropical (subtropical) fruit tree industry development project [(2021)01], and the Hainan University Startup Fund [KYQD(ZR)-20101].

## Conflict of interest

The authors declare that the research was conducted in the absence of any commercial or financial relationships that could be construed as a potential conflict of interest.

## Publisher’s note

All claims expressed in this article are solely those of the authors and do not necessarily represent those of their affiliated organizations, or those of the publisher, the editors and the reviewers. Any product that may be evaluated in this article, or claim that may be made by its manufacturer, is not guaranteed or endorsed by the publisher.

## Footnotes

^1^ https://github.com/Nextomics/NextDenovo

^2^ https://github.com/aidenlab/Juicebox

^3^ http://ccb.jhu.edu/software/hisat2/faq.shtml

^4^ https://github.com/davidemms/OrthoFinder

^5^ https://github.com/broadinstitute/picard

^6^ http://evolution.genetics.washington.edu/phylip.html

^7^ www.timetree.org
