# Supplementary figures and images for: High-quality reference genome sequences of two Cannaceae species provide insights into the evolution of Cannaceae
Source: Front Plant Sci. 2022 Jul 28;13:955904. doi: 10.3389/fpls.2022.955904 (PMC9371203; doi:10.3389/fpls.2022.955904)

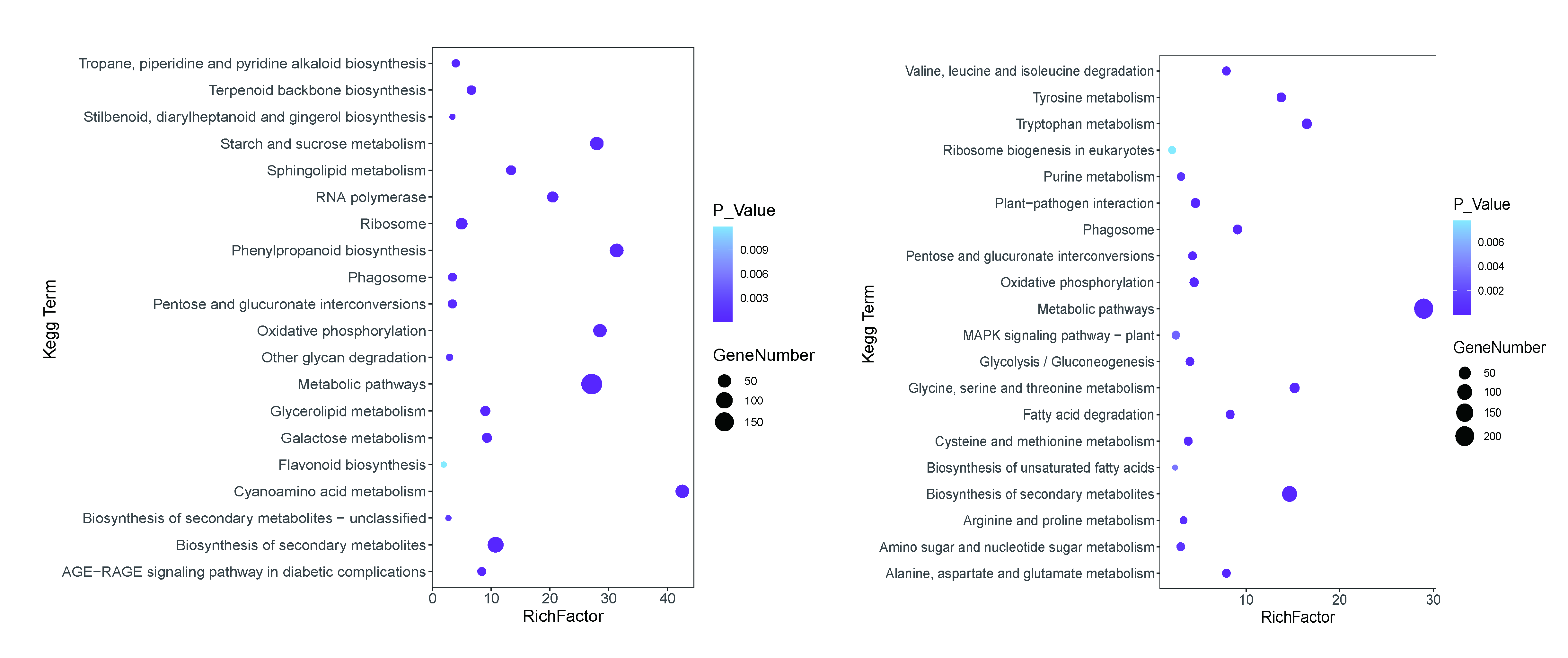

Supplement: Supplementary file 1 [file Image_1.TIFF]

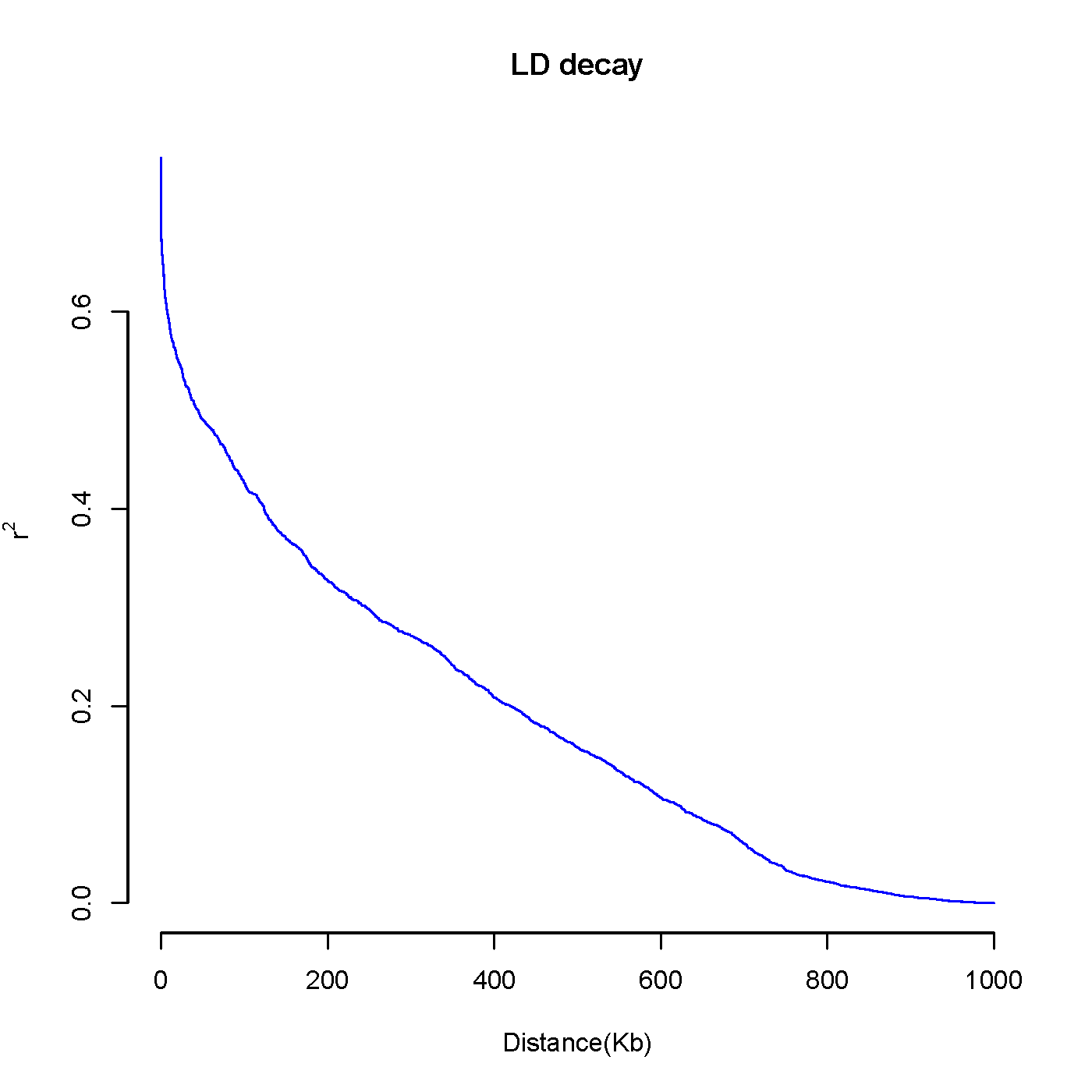

Supplement: Supplementary file 2 [file Image_2.TIFF]

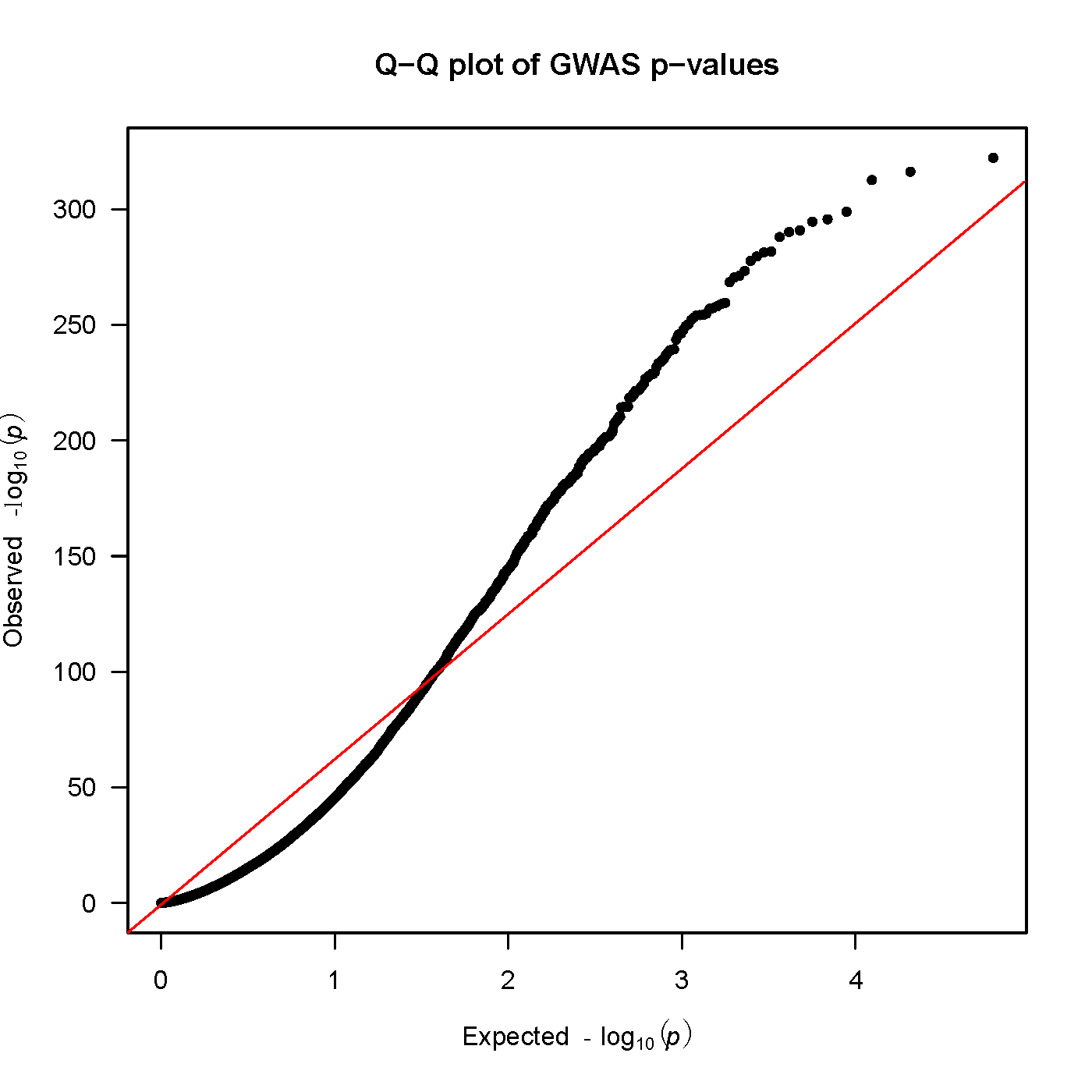

Supplement: Supplementary file 3 [file Image_3.TIFF]

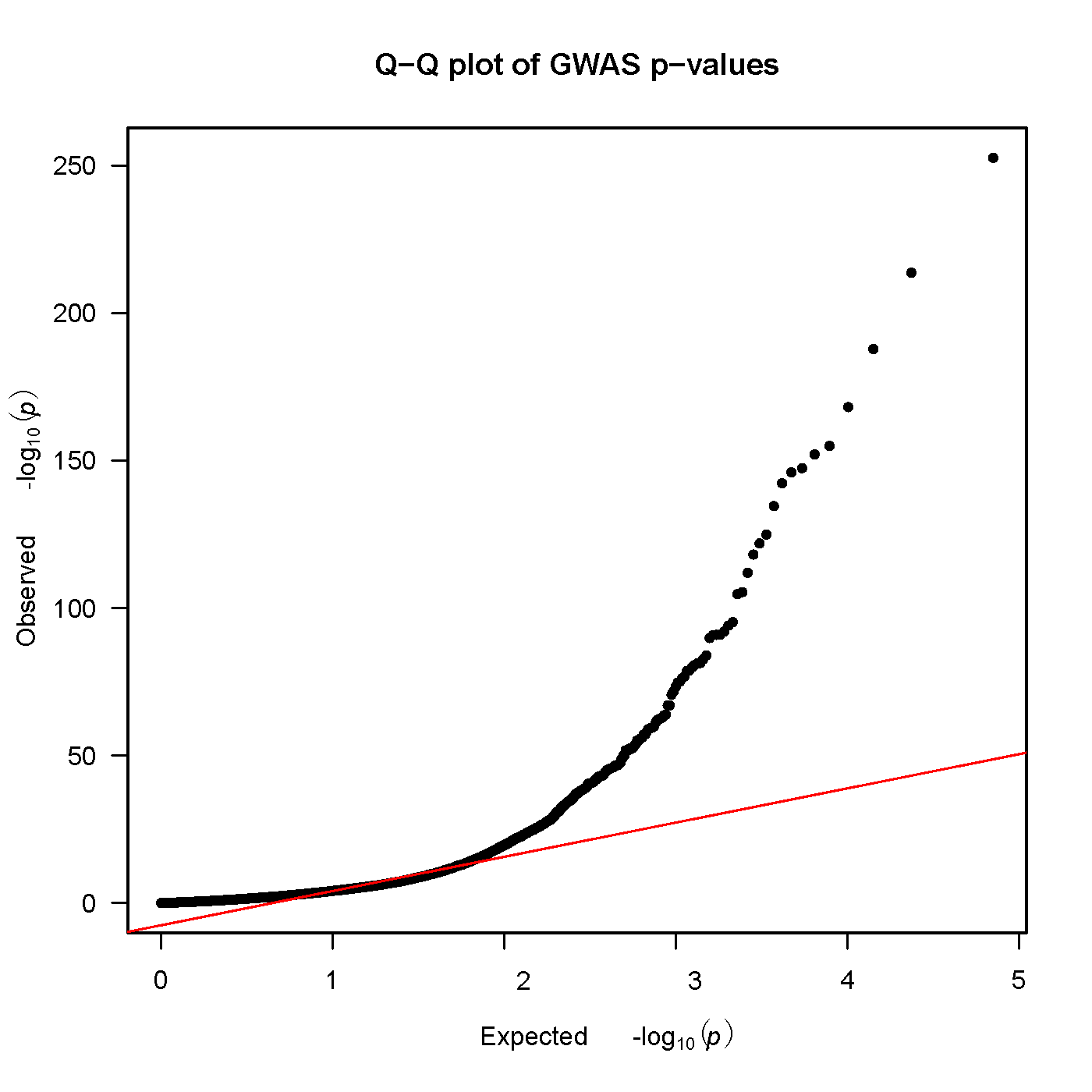

Supplement: Supplementary file 4 [file Image_4.TIFF]
